# An introduction to immunology and immunopathology

**DOI:** 10.1186/s13223-018-0278-1

**Published:** 2018-09-12

**Authors:** Jean S. Marshall, Richard Warrington, Wade Watson, Harold L. Kim

**Affiliations:** 1 Department of Microbiology and Immunology, Dalhousie University, Halifax, NS Canada; 2 Section of Allergy & Clinical Immunology, Department of Internal Medicine, University of Manitoba, Winnipeg, MB Canada; 3 Division of Allergy, Department of Pediatrics, IWK Health Centre, Dalhousie University, Halifax, NS Canada; 4 Western University, London, ON Canada; 5 McMaster University, Hamilton, ON Canada

## Abstract

Beyond structural and chemical barriers to pathogens, the immune system has two fundamental lines of defense: innate immunity and adaptive immunity. Innate immunity is the first immunological mechanism for fighting against an intruding pathogen. It is a rapid immune response, initiated within minutes or hours after aggression, that has no immunologic memory. Adaptive immunity, on the other hand, is antigen-dependent and antigen-specific; it has the capacity for memory, which enables the host to mount a more rapid and efficient immune response upon subsequent exposure to the antigen. There is a great deal of synergy between the adaptive immune system and its innate counterpart, and defects in either system can provoke illness or disease, such as inappropriate inflammation, autoimmune diseases, immunodeficiency disorders and hypersensitivity reactions. This article provides a practical overview of innate and adaptive immunity, and describes how these host defense mechanisms are involved in both heath and illness.

## Background

There are continuous advances in our current understanding of the immune system and how it functions to protect the body from infection. Given the complex nature of this subject, it is beyond the scope of this article to provide an in-depth review of all aspects of immunology. Rather, the purpose of this article is to provide medical students, medical residents, primary-care practitioners and other healthcare professionals with a basic introduction to the main components and function of the immune system and its role in both health and disease. This article will also serve as a backgrounder to the immunopathological disorders discussed in the remainder of this supplement.

## The immune system: innate and adaptive immunity

The immune system refers to a collection of cells, chemicals and processes that function to protect the skin, respiratory passages, intestinal tract and other areas from foreign antigens, such as microbes (organisms such as bacteria, fungi, and parasites), viruses, cancer cells, and toxins. Beyond, the structural and chemical barriers which protect us from infection, the immune system can be simplistically viewed as having two “lines of defense”: innate immunity and adaptive immunity. Innate immunity represents the first line of defense to an intruding pathogen. It is an antigen-independent (non-specific) defense mechanism that is used by the host immediately or within hours of encountering an antigen. The innate immune response has no immunologic memory and, therefore, it is unable to recognize or “memorize” the same pathogen should the body be exposed to it in the future. Adaptive immunity, on the other hand, is antigen-dependent and antigen-specific and, therefore, involves a lag time between exposure to the antigen and maximal response. The hallmark of adaptive immunity is the capacity for memory which enables the host to mount a more rapid and efficient immune response upon subsequent exposure to the antigen. Innate and adaptive immunity are not mutually exclusive mechanisms of host defense, but rather are complementary, with defects in either system resulting in host vulnerability or inappropriate responses [1–3].

### Innate immunity

Innate immunity can be viewed as comprising four types of defensive barriers: anatomic (skin and mucous membrane), physiologic (temperature, low pH and chemical mediators), endocytic and phagocytic, and inflammatory. Table 1 summarizes the non-specific host-defense mechanisms for each of these barriers. Cells and processes that are critical for effective innate immunity to pathogens that evade the anatomic barriers have been widely studied. Innate immunity to pathogens relies on pattern recognition receptors (PRRs) which allow a limited range of immune cells to detect and respond rapidly to a wide range of pathogens that share common structures, known as pathogen associated molecular patterns (PAMPs). Examples of these include bacterial cell wall components such as lipopolysaccharides (LPS) and double-stranded ribonucleic acid (RNA) produced during viral infection.

**Table 1:** Summary of non-specific host-defense mechanisms for barriers of innate immunity [1]

Barrier	Mechanism
**Anatomic**	
Skin	• Mechanical barrier retards entry of microbes • Acidic environment (pH 3–5) retards growth of microbes
Mucous membrane	• Normal flora compete with microbes for attachment sites • Mucous entraps foreign microbes • Cilia propel microbes out of body
**Physiologic**	
Temperature	• Body temperature/fever response inhibits growth of some pathogens
Low pH	• Acidic pH of stomach kills most undigested microbes
Chemical mediators	• Lysozyme cleaves bacterial cell wall • Interferon induces antiviral defenses in uninfected cells • Complement lyses microbes or facilitates phagocytosis
**Phagocytic/endocytic barriers**	
	• Various cells internalize (endocytosis) and break down foreign macromolecules • Specialized cells (blood monocytes, neutrophils, tissue macrophages) internalize (phagocytose), kill and digest whole organisms
**Inflammatory barriers**	
	• Tissue damage and infection induce leakage of vascular fluid containing serum protein with antibacterial activity, leading to influx of phagocytic cells into the affected area

An important function of innate immunity is the rapid recruitment of immune cells to sites of infection and inflammation through the production of cytokines and chemokines (small proteins involved in cell–cell communication and recruitment). Cytokine production during innate immunity mobilizes many defense mechanisms throughout the body while also activating local cellular responses to infection or injury. Key inflammatory cytokines released during the early response to bacterial infection are: tumour necrosis factor (TNF), interleukin 1 (IL-1) and interleukin 6 (IL-6). These cytokines are critical for initiating cell recruitment and the local inflammation which is essential for clearance of many pathogens. They also contribute to the development of fever. Dysregulated production of such inflammatory cytokines is often associated with inflammatory or autoimmune disease, making them important therapeutic targets.

The complement system is a biochemical cascade that functions to identify and opsonize (coat) bacteria and other pathogens. It renders pathogens susceptible to phagocytosis, a process by which immune cells engulf microbes and remove cell debris, and also kills some pathogens and infected cells directly. The phagocytic action of the innate immune response promotes clearance of dead cells or antibody complexes and removes foreign substances present in organs, tissues, blood and lymph. It can also activate the adaptive immune response through the mobilization and activation of antigen-presenting cells (APCs) (discussed later) [1, 3].

**Fig. 1:**
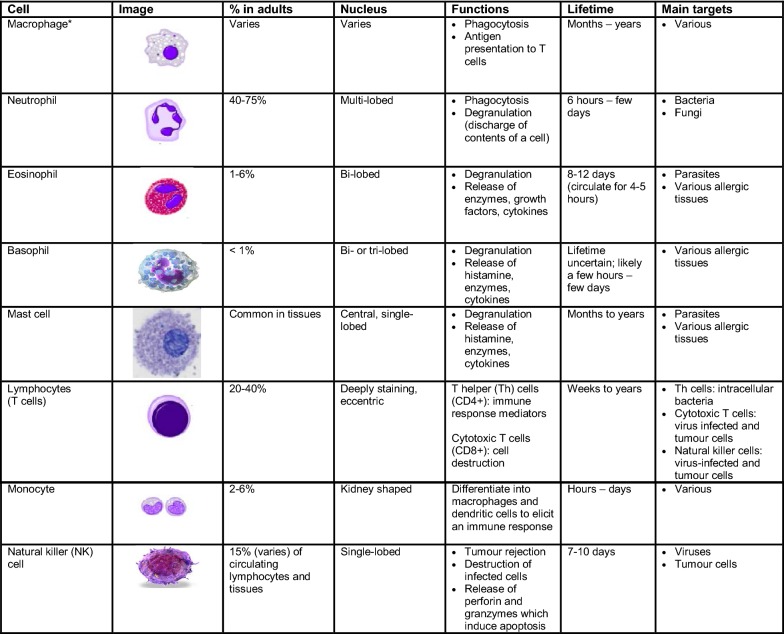
**Characteristics and function of cells involved in innate immunity** [1, 3, 4]. *Dust cells (within pulmonary alveolus), histiocytes (connective tissue), Kupffer cells (liver), microglial cells (neural tissue), epithelioid cells (granulomas), osteoclasts (bone), mesangial cells (kidney)

Numerous cells are involved in the innate immune response such as phagocytes (macrophages and neutrophils), dendritic cells, mast cells, basophils, eosinophils, natural killer (NK) cells and innate lymphoid cells. Phagocytes are sub-divided into two main cell types: neutrophils and macrophages. Both of these cells share a similar function: to engulf (phagocytose) microbes and kill them through multiple bactericidal pathways. In addition to their phagocytic properties, neutrophils contain granules and enzyme pathways that assist in the elimination of pathogenic microbes. Unlike neutrophils (which are short-lived cells), macrophages are long-lived cells that not only play a role in phagocytosis, but are also involved in antigen presentation to T cells (see Fig. 1) [1].

Dendritic cells also phagocytose and function as APCs, initiating the acquired immune response and acting as important messengers between innate and adaptive immunity. Mast cells and basophils share many salient features with each other, and both are instrumental in the initiation of acute inflammatory responses, such as those seen in allergy and asthma. Mast cells also have important functions as immune “sentinel cells” and are early producers of cytokines in response to infection or injury. Unlike mast cells, which generally reside in the connective tissue surrounding blood vessels and are particularly common at mucosal surfaces, basophils reside in the circulation. Eosinophils are granulocytes that possess phagocytic properties and play an important role in the destruction of parasites that are often too large to be phagocytosed. Along with mast cells and basophils, they also control mechanisms associated with allergy and asthma. Natural killer (NK) cells play a major role in the rejection of tumours and the destruction of cells infected by viruses. Destruction of infected cells is achieved through the release of perforins and granzymes (proteins that cause lysis of target cells) from NK-cell granules which induce apoptosis (programmed cell death) [4]. NK cells are also an important source of another cytokine, interferon-gamma (IFN-γ), which helps to mobilize APCs and promote the development of effective anti-viral immunity. Innate lymphoid cells (ILCs) play a more regulatory role. Depending on their type (i.e., ILC-1, ILC-2, ILC-3), they selectively produce cytokines such as IL-4, IFN-γ and IL-17 that help to direct the appropriate immune response to specific pathogens and contribute to immune regulation in that tissue.

The main characteristics and functions of the cells involved in the innate immune response are summarized in Fig. 1.

### Adaptive immunity

The development of adaptive immunity is aided by the actions of the innate immune system, and is critical when innate immunity is ineffective in eliminating infectious agents. The primary functions of the adaptive immune response are: the recognition of specific “non-self” antigens, distinguishing them from “self” antigens; the generation of pathogen-specific immunologic effector pathways that eliminate specific pathogens or pathogen-infected cells; and the development of an immunologic memory that can quickly eliminate a specific pathogen should subsequent infections occur [2]. Adaptive immune responses are the basis for effective immunization against infectious diseases. The cells of the adaptive immune system include: antigen-specific T cells, which are activated to proliferate through the action of APCs, and B cells which differentiate into plasma cells to produce antibodies.

#### T cells and APCs

T cells derive from hematopoietic stem cells in bone marrow and, following migration, mature in the thymus. These cells express a series of unique antigen-binding receptors on their membrane, known as the T-cell receptor (TCR). Each T cell expresses a single type of TCR and has the capacity to rapidly proliferate and differentiate if it receives the appropriate signals. As previously mentioned, T cells require the action of APCs (usually dendritic cells, but also macrophages, B cells, fibroblasts and epithelial cells) to recognize a specific antigen.

The surfaces of APCs express a group of proteins known as the major histocompatibility complex (MHC). MHC are classified as either class I (also termed human leukocyte antigen [HLA] A, B and C) which are found on all nucleated cells, or class II (also termed HLA DP, DQ and DR) which are found only on certain cells of the immune system, including macrophages, dendritic cells and B cells. Class I MHC molecules present endogenous (intracellular) peptides, while class II molecules on APCs present exogenous (extracellular) peptides to T cells. The MHC protein displays fragments of antigens (peptides) when a cell is infected with an intracellular pathogen, such as a virus, or has phagocytosed foreign proteins or organisms [2, 3].

**Fig. 2:**
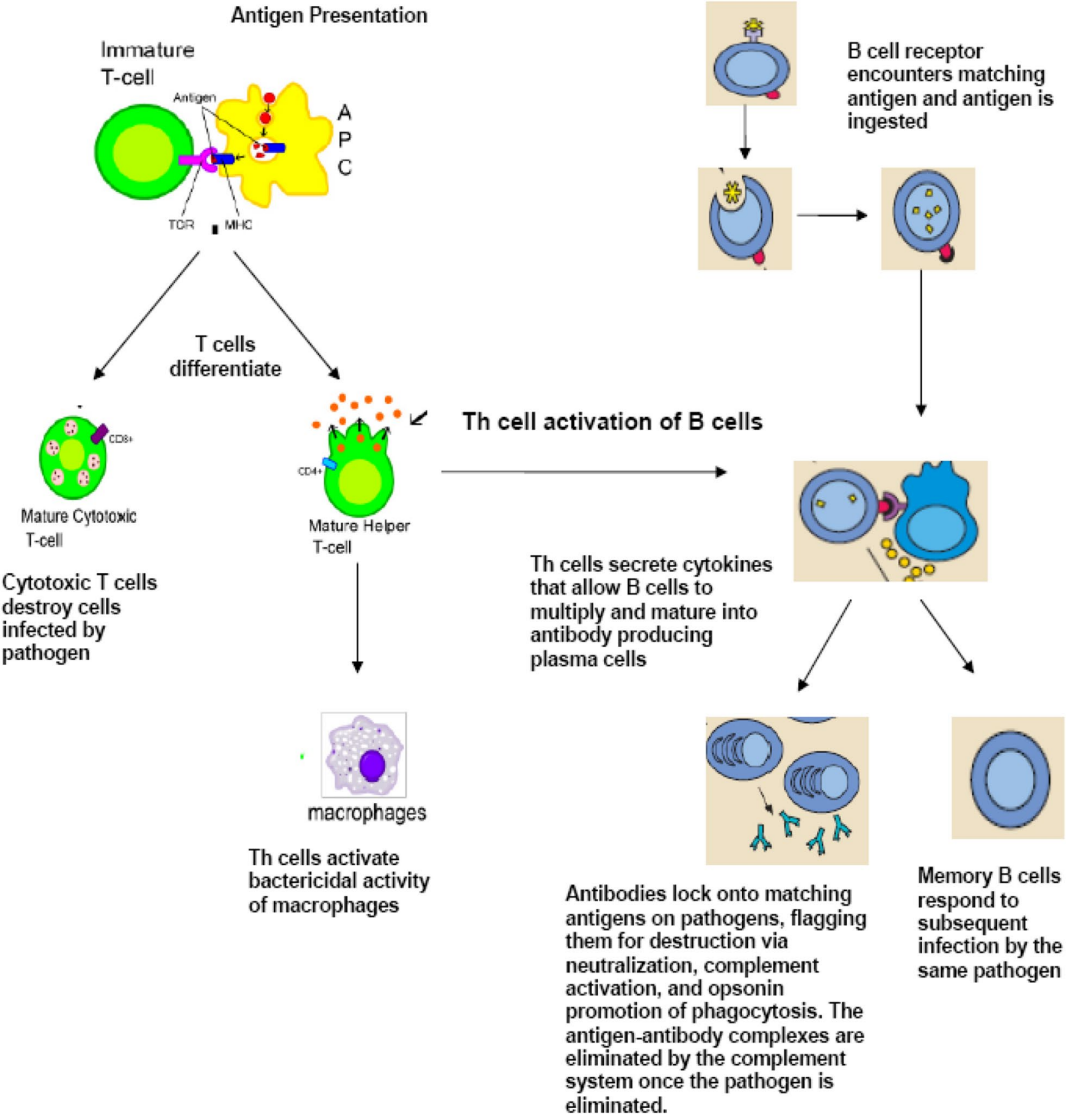
**Adaptive immunity: T-cell and B-cell activation and function.** *APC* antigen-presenting cell, *TCR* T-cell receptor, *MHC* major histocompatibility complex (figure adapted from images available at: http://en.wikipedia.org/wiki/Image:B_cell_activation.png and http://commons.wikimedia.org/wiki/Image:Antigen_presentation.svg)

T cells have a wide range of unique TCRs which can bind to specific foreign peptides. During the development of the immune system, T cells that would react to antigens normally found in our body are largely eliminated. T cells are activated when they encounter an APC that has digested an antigen and is displaying the correct antigen fragments (peptides) bound to its MHC molecules. The opportunities for the right T cells to be in contact with an APC carrying the appropriate peptide MHC complex are increased by the circulation of T cells throughout the body (via the lymphatic system and blood stream) and their accumulation (together with APCs) in lymph nodes. The MHC-antigen complex activates the TCR and the T cell secretes cytokines which further control the immune response. This antigen presentation process stimulates T cells to differentiate primarily into either cytotoxic T cells (CD8+ cells) or T-helper (Th) cells (CD4+ cells) (see Fig. 2). CD8+ cytotoxic T cells are primarily involved in the destruction of cells infected by foreign agents, such as viruses, and the killing of tumour cells expressing appropriate antigens. They are activated by the interaction of their TCR with peptide bound to MHC class I molecules. Clonal expansion of cytotoxic T cells produces effector cells which release substances that induce apoptosis of target cells. Upon resolution of the infection, most effector cells die and are cleared by phagocytes. However, a few of these cells are retained as memory cells that can quickly differentiate into effector cells upon subsequent encounters with the same antigen [2, 3].

CD4+ Th cells play an important role in establishing and maximizing the immune response. These cells have no cytotoxic or phagocytic activity, and cannot directly kill infected cells or clear pathogens. However, they “mediate” the immune response by directing other cells to perform these tasks and regulate the type of immune response that develops. Th cells are activated through TCR recognition of antigen bound to class II MHC molecules. Once activated, Th cells release cytokines that influence the activity of many cell types, including the APCs that activate them.

Several types of Th cell responses can be induced by an APC, with Th1, Th2 and Th17 being the most frequent. The Th1 response is characterized by the production of IFN-γ which activates the bactericidal activities of macrophages and enhances anti-viral immunity as well as immunity to other intracellular pathogens. Th1-derived cytokines also contribute to the differentiation of B cells to make opsonizing antibodies that enhance the efficiency of phagocytes. An inappropriate Th1 response is associated with certain autoimmune diseases.

The Th2 response is characterized by the release of cytokines (IL-4, 5 and 13) which are involved in the development of immunoglobulin E (IgE) antibody-producing B cells, as well as the development and recruitment of mast cells and eosinophils that are essential for effective responses against many parasites. In addition, they enhance the production of certain forms of IgG that aid in combatting bacterial infection. As mentioned earlier, mast cells and eosinophils are instrumental in the initiation of acute inflammatory responses, such as those seen in allergy and asthma. IgE antibodies are also associated with allergic reactions (see Table 2). Therefore, an imbalance of Th2 cytokine production is associated with the development of atopic (allergic) conditions. Th17 cells have been more recently described. They are characterized by the production of cytokines of the IL-17 family, and are associated with ongoing inflammatory responses, particularly in chronic infection and disease. Like cytotoxic T cells, most Th cells will die upon resolution of infection, with a few remaining as Th memory cells [2, 3].

**Table 2:** Major functions of human Ig antibodies [5]

Ig antibody	Function
IgM	• First immunoglobulin (Ig) expressed during B cell development (primary response; early antibody) • Opsonizing (coating) antigen for destruction • Complement fixation
IgG	• Main Ig during secondary immune response • Only antibody capable of crossing the placental barrier • Neutralization of toxins and viruses • Opsonizing (coating) antigen for destruction • Complement fixation
IgD	• Function unclear; appears to be involved in homeostasis
IgA	• Mucosal response; protects mucosal surfaces from toxins, viruses and bacteria through either direct neutralization or prevention of binding to mucosal surface
IgE	• Associated with hypersensitivity and allergic reactions • Plays a role in immune response to parasites

A subset of the CD4+ T cell, known as the regulatory T cell (T reg), also plays a role in the immune response. T reg cells limit and suppress immune responses and, thereby, may function to control aberrant responses to self-antigens and the development of autoimmune disease. T reg cells may also help in the resolution of normal immune responses, as pathogens or antigens are eliminated. These cells also play a critical role in the development of “immune tolerance” to certain foreign antigens, such as those found in food.

#### B cells

B cells arise from hematopoietic stem cells in the bone marrow and, following maturation, leave the marrow expressing a unique antigen-binding receptor on their membrane. Unlike T cells, B cells can recognize antigens directly, without the need for APCs, through unique antibodies expressed on their cell surface. The principal function of B cells is the production of antibodies against foreign antigens which requires their further differentiation [2, 3]. Under certain circumstances, B cells can also act as APCs.

When activated by foreign antigens to which they have an appropriate antigen specific receptor, B cells undergo proliferation and differentiate into antibody-secreting plasma cells or memory B cells (see Fig. 2). Memory B cells are “long-lived” survivors of past infection and continue to express antigen-binding receptors. These cells can be called upon to respond quickly by producing antibodies and eliminating an antigen upon re-exposure. Plasma cells, on the other hand, are relatively short-lived cells that often undergo apoptosis when the inciting agent that induced the immune response is eliminated. However, these cells produce large amounts of antibody that enter the circulation and tissues providing effective protection against pathogens.

Given their function in antibody production, B cells play a major role in the humoral or antibody-mediated immune response (as opposed to the cell-mediated immune response, which is governed primarily by T cells) [2, 3].

## Antibody-mediated vs. cell-mediated immunity

Antibody-mediated immunity is the branch of the acquired immune system that is mediated by B-cell-antibody production. The antibody-production pathway begins when the B cell’s antigen-binding receptor recognizes and binds to antigen in its native form. Local Th cells secrete cytokines that help the B cell multiply and direct the type of antibody that will be subsequently produced. Some cytokines, such as IL-6, help B-cells to mature into antibody-secreting plasma cells. The secreted antibodies bind to antigens on the surface of pathogens, flagging them for destruction through complement activation, opsonin promotion of phagocytosis and pathogen elimination by immune effector cells. Upon elimination of the pathogen, the antigen–antibody complexes are cleared by the complement cascade (see Fig. 2) [2].

Five major types of antibodies are produced by B cells: IgA, IgD, IgE, IgG and IgM. IgG antibodies can be further subdivided into structurally distinct subclasses with differing abilities to fix complement, act as opsonins, etc. The major classes of antibodies have substantially different biological functions and recognize and neutralize specific pathogens. Table 2 summarizes the various functions of the five Ig antibodies [5].

Antibodies play an important role in containing virus proliferation during the acute phase of infection. However, they are not generally capable of eliminating a virus once infection has occurred. Once an infection is established, cell-mediated immune mechanisms are most important in host defense against most intracellular pathogens.

Cell-mediated immunity does not involve antibodies, but rather protects an organism through [2]:

- The activation of antigen-specific cytotoxic T cells that induce apoptosis of cells displaying foreign antigens or derived peptides on their surface, such as virus-infected cells, cells with intracellular bacteria, and cancer cells displaying tumour antigens;
- The activation of macrophages and NK cells, enabling them to destroy intracellular pathogens; and
- The stimulation of cytokine (such as IFNγ) production that further mediates the effective immune response.

Cell-mediated immunity is directed primarily at microbes that survive in phagocytes as well as those that infect non-phagocytic cells. This type of immunity is most effective in eliminating virus-infected cells and cancer cells, but can also participate in defending against fungi, protozoa, cancers, and intracellular bacteria. Cell-mediated immunity also plays a major role in transplant rejection.

## Passive vs. active immunization

Acquired immunity is attained through either passive or active immunization. Passive immunization refers to the transfer of *active* humoral immunity, in the form of “ready-made” antibodies, from one individual to another. It can occur naturally by transplacental transfer of maternal antibodies to the developing fetus, or it can be induced artificially by injecting a recipient with exogenous antibodies that are usually manufactured for this purpose and that are targeted to a specific pathogen or toxin. The latter is used when there is a high risk of infection and insufficient time for the body to develop its own immune response, or to reduce the symptoms of chronic or immunosuppressive diseases.

Active immunization refers to the production of antibodies against a specific antigen or pathogen *after* exposure to the antigen. It can be acquired through either natural infection with a microbe or through administration of a vaccine that can consist of attenuated (weakened) pathogens, inactivated organisms or specific proteins or carbohydrates known to induce immunity. Effective active immunization often requires the use of “adjuvants” which improve the ability of the immune system to respond to antigen injection.

## Immunopathology

As mentioned earlier, defects or malfunctions in either the innate or adaptive immune response can provoke illness or disease. Such disorders are generally caused by an overactive immune response (known as hypersensitivity reactions), an inappropriate reaction to self (known as autoimmunity) or ineffective immune responses (known as immunodeficiency).

### Hypersensitivity reactions

Hypersensitivity reactions refer to undesirable responses produced by the normal immune system. There are four types of hypersensitivity reactions [6, 7]:

- Type I: immediate hypersensitivity.
- Type II: cytotoxic or antibody-dependent hypersensitivity.
- Type III: immune complex disease.
- Type IV: delayed-type hypersensitivity.

Type I hypersensitivity is the most common type of hypersensitivity reaction. It is an allergic reaction provoked by re-exposure to a specific type of antigen, referred to as an allergen. Unlike the normal immune response, the type I hypersensitivity response is characterized by the secretion of IgE by plasma cells. IgE antibodies bind to receptors on the surface of tissue mast cells and blood basophils, causing them to be “sensitized”. Later exposure to the same allergen cross-links the bound IgE on sensitized cells resulting in degranulation and the secretion of active mediators such as histamine, leukotrienes, and prostaglandins that cause vasodilation and smooth-muscle contraction of the surrounding tissue. Common environmental allergens inducing IgE-mediated allergies include pet (e.g., cat, dog, horse) epithelium, pollen, house dust mites, and molds. Food allergens are also a common cause of type I hypersensitivity reactions, however, these types of reactions are more frequently seen in children than adults. Treatment of type I reactions generally involves trigger avoidance, and in the case of inhaled allergens, pharmacological intervention with bronchodilators, antihistamines and anti-inflammatory agents. Some types of allergic disease can be treated with immunotherapy (see *Allergen-specific Immunotherapy* article in this supplement). Severe cases of type 1 hypersensitivity (anaphylaxis) may require immediate treatment with epinephrine.

Type II hypersensitivity reactions are rare and take anywhere from 2 to 24 h to develop. These types of reactions occur when IgG and IgM antibodies bind to the patient’s own cell-surface molecules, forming complexes that activate the complement system. This, in turn, leads to opsonization, red blood cell agglutination (process of agglutinating or “clumping together”), cell lysis and death. Some examples of type II hypersensitivity reactions include: erythroblastosis fetalis, Goodpasture syndrome, and autoimmune anemias.

Type III hypersensitivity reactions occur when IgG and IgM antibodies bind to soluble proteins (rather than cell surface molecules as in type II hypersensitivity reactions) forming immune complexes that can deposit in tissues, leading to complement activation, inflammation, neutrophil influx and mast cell degranulation. This type of reaction can take days, or even weeks, to develop and treatment generally involves anti-inflammatory agents and corticosteroids. Examples of type III hypersensitivity reactions include systemic lupus erythematosus (SLE), serum sickness and reactive arthritis.

Unlike the other types of hypersensitivity reactions, type IV reactions are cell-mediated and antibody-independent. They are the second most common type of hypersensitivity reaction and usually take 2 or more days to develop. These types of reactions are caused by the overstimulation of T cells and monocytes/macrophages which leads to the release of cytokines that cause inflammation, cell death and tissue damage. In general, these reactions are easily resolvable through trigger avoidance and the use of topical corticosteroids. An example of this is the skin response to poison ivy.

A brief summary of the four types of hypersensitivity reactions is provided in Table 3.

**Table 3:** Types of hypersensitivity reactions [6, 7]

Type	Alternate name	Examples	Mediators
I	Allergy (immediate)	• Atopy – Anaphylaxis – Asthma – Allergic rhinitis – Angioedema – Food allergy	IgE
II	Cytotoxic, antibody-dependent	• Erythroblastosis fetalis • Goodpasture syndrome • Autoimmune anemias, thrombocytopenias	IgG, IgM
III	Immune complex disease	• Systemic lupus erythematosus • Serum sickness • Reactive arthritis • Arthrus reaction	Aggregation of antigens IgG, IgM Complement proteins
IV	Delayed-type hypersensitivity, cell-mediated, antibody-independent	• Contact dermatitis • Tuberculosis • Chronic transplant rejection	T cells, monocytes, macrophages

### Autoimmunity

Autoimmunity involves the loss of normal immune homeostasis such that the organism produces an abnormal response to its own tissue. The hallmark of autoimmunity is the presence of self-reactive T cells, auto-antibodies, and inflammation. Prominent examples of autoimmune diseases include: Celiac disease, type 1 diabetes mellitus, Addison’s disease and Graves’ disease [8].

### Inflammation

Poorly regulated inflammatory responses and tissue damage as a result of inflammation are often immunopathological features. Defects in immune regulation are associated with many chronic inflammatory diseases, including: rheumatoid arthritis, psoriasis, inflammatory bowel disease and asthma. Classical features of inflammation are heat, redness, swelling and pain. Inflammation can be part of the normal host response to infection and a required process to rid the body of pathogens, or it may become uncontrolled and lead to chronic inflammatory disease. The overproduction of inflammatory cytokines (such as TNF, IL-1 and IL-6) as well as the recruitment of inflammatory cells (such as neutrophils and monocytes) through the function of chemokines are important drivers of the inflammatory process. Additional mediators produced by recruited and activated immune cells induce changes in vascular permeability and pain sensitivity.

### Immunodeficiency

Immunodeficiency refers to a state in which the immune system’s ability to fight infectious disease is compromised or entirely absent. Immunodeficiency disorders may result from a primary genetic defect (primary immunodeficiency—see *Primary Immunodeficiency* article in this supplement) which can effect either innate or acquired immune function through inhibition of selected immune cells or pathways, or it may be acquired from a secondary cause (secondary immunodeficiency), such as viral or bacterial infections, malnutrition, autoimmunity or treatment with drugs that induce immunosuppression. Certain diseases can also directly or indirectly impair the immune system such as leukemia and multiple myeloma. Immunodeficiency is also the hallmark of acquired immunodeficiency syndrome (AIDS), caused by the human immunodeficiency virus (HIV). HIV directly infects Th cells and also impairs other immune system responses indirectly [9, 10].

## Conclusion

Innate immunity is the first immunological, non-specific mechanism for fighting against infections. This immune response is rapid, occurring minutes or hours after aggression and is mediated by numerous cells including phagocytes, mast cells, basophils and eosinophils, as well as the complement system. Adaptive immunity develops in conjunction with innate immunity to eliminate infectious agents; it relies on the tightly regulated interplay between T cells, APCs and B cells. A critical feature of adaptive immunity is the development of immunologic memory or the ability of the system to learn or record its experiences with various pathogens, leading to effective and rapid immune responses upon subsequent exposure to the same or similar pathogens. A brief overview of the defining features of innate and adaptive immunity are presented in Table 4.

**Table 4:** Overview of the defining features of innate and adaptive immunity [1]

	Innate immune system	Adaptive immune system
Cells	Hematopoietic cells: • Macrophages • Dendritic cells • Mast cells • Neutrophils • Basophils • Eosinophils • NK cells • T cells Non-hematopoietic cells • Epithelial cells (skin, airways, gastrointestinal tract)	Hematopoietic cells: • T cells • B cells
Molecules	• Cytokines • Complement • Proteins and glycoprotein	• Antibodies (Ig) • Cytokines
Response time	• Immediate	• Delayed by hours to days
Immunologic memory	• None: responses are the same with each exposure	• Responsiveness enhanced by repeated antigen exposure

There is a great deal of synergy between the adaptive immune system and its innate counterpart, and defects in either system can lead to immunopathological disorders, including autoimmune diseases, immunodeficiencies and hypersensitivity reactions. The remainder of this supplement will focus on the appropriate diagnosis, treatment and management of some of these more prominent disorders, particularly those associated with hypersensitivity reactions.

## Abbreviations

PRRs: pattern recognition receptors
PAMPs: pathogen associated molecular patterns
LPS: lipopolysaccharides
RNA: ribonucleic acid
TNF: tumour necrosis factor
IL: interleukin
APCs: antigen-presenting cells
NK: natural killer
IFN-γ: interferon-gamma
ILCs: innate lymphoid cells
TCR: T cell receptor
MHC: major histocompatibility complex
HLA: human leukocyte antigen
Ig: immunoglobulin
T reg: regulatory T cell
SLE: systemic lupus erythematosus
AIDS: acquired immunodeficiency syndrome
HIV: human immunodeficiency virus
